# Effectiveness of Essence of Chicken on Cognitive Function Improvement: A Randomized Controlled Clinical Trial

**DOI:** 10.3390/nu10070845

**Published:** 2018-06-29

**Authors:** Panrapee Suttiwan, Pongsak Yuktanandana, Sakkaphat Ngamake

**Affiliations:** 1 Faculty of Psychology, Chulalongkorn University, Bangkok 10330, Thailand; cpanrapee@yahoo.com (P.S.); nsakkaphat@gmail.com (S.N.); 2 Faculty of Medicine, Chulalongkorn University, Bangkok 10330, Thailand

**Keywords:** essence of chicken, chicken extract, chicken essence, health-nutritional supplement, cognitive function, stress, memory, working memory, short-term memory, attention

## Abstract

High-quality, adequately-powered clinical trials investigating the effect of Essence of Chicken (EC) on cognitive function are lacking. We conducted a randomized, double-blind, placebo-controlled clinical trial on healthy adult volunteers to determine the effect of EC on short-term memory, working memory, and selective and sustained attention. As a secondary objective, we evaluated baseline stress as a modifying factor by including treatment, stress and visit as main effects in a three-way ANOVA model. Cognitive function was evaluated at baseline, and Days 7 and 14. Data from 235 participants were analyzed on a per-protocol basis. The three-way interaction effect was significant (*p* = 0.020) in Digit Span Forward and further analyses showed EC improved test performance in moderate (*p* = 0.041) and severe stress (*p* = 0.065) but not in normal and mild stress subgroups. In Digit Span Backward, EC group showed greater improvement compared to placebo (*p* = 0.028), with 0.60 digits (8.50% improvement from baseline) more recalled on Day 7. No treatment or interaction effects were statistically significant in selective and sustained attention tests. Our findings support EC’s effect in improving mental processes used in working memory among healthy adults and short-term memory among healthy adults experiencing stress in daily life.

## 1. Introduction

Essence of chicken (EC) is a commonly used health-nutritional supplement in Southeast Asia. It is a liquid supplement made from high-temperature and high-pressure extraction of whole chicken, and is used as a traditional remedy for several ailments. Its uses include improving physical qualities of athletes, providing nutrition for recovering patients and restoring strength to women following childbirth [1]. Scientifically, beneficial effects of EC on physical health in thermic response [2], resting metabolic rate [3], reduction in blood glucose levels and glycemic response [4,5], colostrum composition [6] and recovery from physical exhaustion [7] have been demonstrated in human clinical studies. Beneficial effects on mental health include reduced anxiety [8,9], reduced depression [8,10] and improved general mental health [11] in normal or stressed healthy adults, as well as patients diagnosed with anxiety disorder [9]. EC has also been shown to promote recovery from mental fatigue [10,12,13].

Mental effects of EC in numerous areas of cognitive function have also been studied in clinical trials. These domains include attention (simple, sustained and selective), memory (short-term and working memory) and executive function, with heterogeneous findings reported across studies. Although individual studies have reported improvement in short-term memory [11,13,14], working memory [11,14,15], mental arithmetic [11,13,14] and reaction times [12] due to EC consumption, a systematic review and meta-analysis found large uncertainties on effect sizes and availability of only a small number of low quality trials. The authors concluded that more high-quality randomized control trials (RCTs) were needed to determine EC’s effect on cognitive function [16]. Two other recent studies have since been published [15,17]. Improvement in working memory, decision time and reaction time, but not episodic memory and sustained attention were found with EC consumption in one study [15]. In the other study, no effect of EC on sustained attention, executive function, short-term or working memory was shown among young adults experiencing work stress. However, subgroup analyses showed that EC improved short-term memory in high anxiety and high depression subgroups [17]. 

Physiological and biochemical changes associated with cognitive effects observed during EC consumption in humans include improved blood flow to the brain during a working memory task [18] and faster recovery of blood cortisol levels [13,15], which may benefit cognition as stress is known to be detrimental to cognition [19]. EC contains many different components including proteins, peptides and free amino acids [1] and which or whether multiple components contribute to its cognitive effect has not been fully elucidated. Among these components, carnosine and anserine, which are dipeptides found in EC [20], have shown to preserve or improve episodic memory [21,22,23] and mental status [24] among healthy middle-aged and elderly adults when supplemented orally in other preparations of chicken meat extract. In these studies, preservation of blood flow to the brain [22] and suppression of blood inflammatory chemokines were observed and postulated as mechanisms for the preservation of cognitive function [21,22]. 

Notably, the healthy but stressed adult population has been of special focus in studies of EC effectiveness in cognitive function enhancement. Chronic stress adversely affects cognitive function, with chronic exposure to high levels of glucocorticoids (primarily cortisol in humans) associated with impaired cognitive performance [18]. Faster recovery of serum cortisol levels following acute stressors in healthy adults consuming EC has been previously shown, hence cortisol regulation could be a potential mechanism of EC’s effect on cognitive function [13,15]. Nevertheless, results are somewhat heterogeneous even among the subset of studies involving stressed adults. EC improved short-term memory, working memory and mental arithmetic in two studies involving stressed medical students [11,14], yet no improvement was seen in another study of young adults experiencing work stress [17]. Furthermore, the effect of stress on EC’s effectiveness in improving cognitive function has not been formally evaluated as each of these studies recruited only stressed volunteers. 

Due to the heterogeneous results and lack of high quality, adequately-powered clinical trials [16], our primary study objective was to determine the effect of EC on cognitive function in healthy adults aged 18–45 years old using a high quality randomized clinical trial design. Our secondary objective was to understand the effect of stress in daily life on the potential cognitive-enhancing effects of EC. Specifically, we hypothesize that among participants experiencing more stress in daily life, the cognitive benefits of EC consumption will be larger.

## 2. Materials and Methods 

### 2.1. Study Design

This single center, randomized, placebo-controlled, double-blind, parallel group trial was approved by the Research Ethics Review Committee for Research Involving Human Research Participants, Health Sciences Group, Chulalongkorn University, Thailand (Study No. 010.1/59) prior to commencement. All subjects gave informed consent for inclusion before enrolment into the study which was conducted in accordance with the Declaration of Helsinki. After enrolment, participants were assigned identification numbers (ID) in ascending order and randomly allocated in equal proportions to three groups: placebo, one bottle/day of EC and two bottles/day of EC using a blocked randomized list. The study sponsor labeled all investigational products with participant IDs before study commencement, and the study site team received a blinded list of participant IDs. Participants and investigators were blinded to the treatment assignment. Participants were instructed to consume one bottle (70 mL) of the study product twice a day at the same time in the morning and evening throughout the 14-day study period. The two bottles/day EC and placebo groups consumed two bottles of essence of chicken or placebo daily, respectively, whereas the one bottle/day EC group consumed one bottle of essence of chicken in the morning and one bottle of placebo at night. Participants attended a total of three study visits, on Days 0 (baseline), 7 and 14. Cognitive function was assessed at each of these visits.

### 2.2. Participants

Participants were recruited using a snowball (non-probabilistic) sampling method. Study inclusion criteria were: 18–45 years old, normal Body Mass Index (BMI) of 18.5–24.9 kg/m^2^ across all ages, no continuous consumption (i.e., regular or everyday consumption) of EC in the preceding three months, and a Chalder Fatigue Questionnaire (CFQ) [25] bimodal score of ≥4, indicating the presence of self-reported fatigue [19]. Individuals with self-reported chronic, malignant disease (e.g., cancer, heart, liver, renal, or other metabolic diseases), psychiatric or neurological diseases were excluded from the study. Pregnant and lactating women, professional athletes, individuals who reported recent lifestyle changes (including diet, e.g., adopting a vegetarian diet, weight loss plan, physical activity, and alcohol consumption/smoking changes), history of allergy to chicken or seafood, or individuals enrolled in other studies were excluded from the study. Written informed consent was obtained from all study participants. Enrolled participants visited the study site at the Faculty of Psychology, Chulalongkorn University, Thailand at screening, Days 0 (baseline), 7 and 14.

To determine baseline stress levels of participants, participants completed the seven-item stress scale of the Depression Anxiety Stress Scales (DASS) [20]. DASS scales have previously shown good internal consistency, convergent and discriminant validity in previous studies [26,27]. In this study, adequate internal consistency was found on the stress scale with a Cronbach’s alpha coefficient of 0.82. For each question, a four-point rating scale is used. The interpretation of summed scores is: “Normal” (score 0–14), “Mild” (score 15–18), “Moderate” (score 19–25), “Severe” (score 26–33) and “Extremely severe” (score +34) stress [28]. Four subgroups were defined in the subgroup analysis according to this standard classification, except for the “Severe” and “Extremely severe” groups which were combined due to low participant numbers in the “Extremely severe” category. 

### 2.3. Investigational Product 

EC used in this study was a commercially available preparation. Both EC and placebo products were provided by Cerebos Pacific Limited trading as BRAND’S Suntory Asia. A bottle of EC (70 mL) contains 5.81 g of protein and peptides. In terms of total amino acid content, the three most abundant amino acids in EC are glutamic acid (642 mg), glycine (541 mg) and arginine (407 mg) [4]. EC also contains 56 mg of hexose, 28 mg of fat, and 3 mg caramel [1], and the di-peptides β-alanyl-l-histidine (carnosine) and β-alanyl-l-methyl-l-histidine (anserine) [20]. EC is produced via a water extraction process from chicken meat for several hours under high-temperature, followed by centrifugation to remove fat and cholesterol, vacuum concentration, and sterilization by high temperature and pressure before bottling. This processing enriches chicken essence to give a protein product that is low in sugar and fat, conveniently available for easy consumption and household storage [23]. Placebo was formulated with marine collagen to have a similar appearance, taste, caloric and protein content to EC (Table 1). Each bottle (70 mL) contained marine collagen 5.60 g, yeast extract (Springer^®^ 2000) 0.30 g, caramel type 1 (520) 0.21 g, NaHCO_3_ 0.23 g, citric acid 0.18 g and water 63.48 g. Comparing EC and placebo formulations, placebo is slightly richer in protein (9.32 g in placebo and 8.02 g in EC) and carbohydrate (0.76 g in placebo and 0.00 g in EC) content. Therefore, any superior cognitive enhancement of EC over placebo shown is likely due to various active components rather than overall macronutrient profile of EC, which has been found to be abundant in amino acids and dipeptides carnosine and anserine [1].

### 2.4. Cognitive Function Assessment 

Administration of the assessments to the participants was by trained research assistants who were graduate students in psychology and/or degree-holders in psychology and blinded to the treatment assignment. Research assistants were trained by P.S., a psychologist and study co-investigator. A computerized cognitive test battery comprising of the Wechsler Adult Intelligence Scale (WAIS) Digit Span test for assessing short-term and working memory [23,24], the Stroop Color–Word test for assessing selective attention [29,30] and the Sustained Attention to Response task (SART) [31] for assessing sustained attention was administered. 

#### 2.4.1. WAIS Digit Span 

Both the WAIS Digit Span Forward and Backward tasks were administered. Digit Span Forward is a measure of short-term memory capacity and Digit Span Backward, which requires mental processing of information to reverse the order of digits before recall, is a measure of working memory capacity [27,28]. In the Forward task, a sequence of auditory digits was played on a computer and participants were asked to recall the digit sequence in order. In the Backward task, participants were asked to recall the digits in the reverse order [32]. 

#### 2.4.2. Stroop Color–Word Test

The Stroop Color–Word test measures selective attention, specifically, the ability to inhibit cognitive interference and mental flexibility [30]. In the Stroop Color–Word test (incongruous condition), participants are asked to indicate the color in which each word is printed in, while ignoring the meaning of the words. The difficulty in inhibiting the more automated process (reading of words) is called the Stroop effect [33]. Performance in this task relies on concentration, attention and a specific executive-frontal domain function to continuously block out the automatic processing of reading [34]. 

#### 2.4.3. Sustained Attention to Response Task (SART) 

The Sustained Attention to Response Task (SART) measures failure of sustained attention [35]. Participants were first shown a single digit, 1–9, on a screen in varying font sizes. The digit then disappears and is replaced with a mask (circle with an “X”). Participants are asked to press the spacebar if any digit other than 3 is presented, and to withhold the response if digit 3 presented. A distinctive feature of the SART is that the automatic response is the “default” condition (i.e., pressing the spacebar) that must be periodically overridden by a conscious, executive decision (i.e., to withhold pressing the spacebar) [31]. The failure to note the no-go signal indicates the failure of sustained attention. 

### 2.5. Sample Size Calculations 

The target sample size was 115 participants per treatment group, based on a two-sided alpha of 0.05, 80% statistical power, effect size of 0.375 and anticipated participant attrition of 15%. The effect size was estimated from standardized mean differences calculated from previous EC studies using digit span tests [11,14] and arrow-flankers test (measuring selective attention) [15].

### 2.6. Statistical Analysis 

Demographic and baseline characteristics are compared between EC and placebo groups in Table 2. Cognitive function test parameters were analyzed using a three-way repeated measures analysis of variance (ANOVA) of change scores (outcome–baseline score). In the three-way ANOVA model, treatment, visit and stress at baseline (DASS categories: normal, mild, moderate and severe stress) were included as main effects. Three two-way interaction terms and one three-way interaction term were also included. The three-way interaction term (treatment × stress × visit), when statistically significant, indicates that the treatment effect of EC is different across subgroups. The primary analysis of treatment effectiveness on cognitive function was per-protocol among participants who completed the study, whereas safety data were analyzed for enrolled participants. The data analysis for this paper was generated using SAS software, Version 9.4 of the SAS^®^ System for Windows [36] and IBM SPSS Statistics for Windows, Version 22.0.

### 2.7. Protocol Deviations

Although the trial was conducted as a three-arm study, only results from the two bottles/day EC and placebo arms are presented in this paper due to anomalous trends found in the one bottle/day EC arm during data analysis. Investigation of subject diaries for potential reasons suggest that accidental unblinding could have occurred among the one bottle/day EC arm, as five participants recorded a taste difference between the morning and evening preparations in their subject diaries. As interpretation of this study arm’s results would be uncertain, only the results from the two bottles/day EC and placebo arms were included in the final analysis and presentation in this paper. 

## 3. Results

### 3.1. Participant Demographics

**Figure 1:**
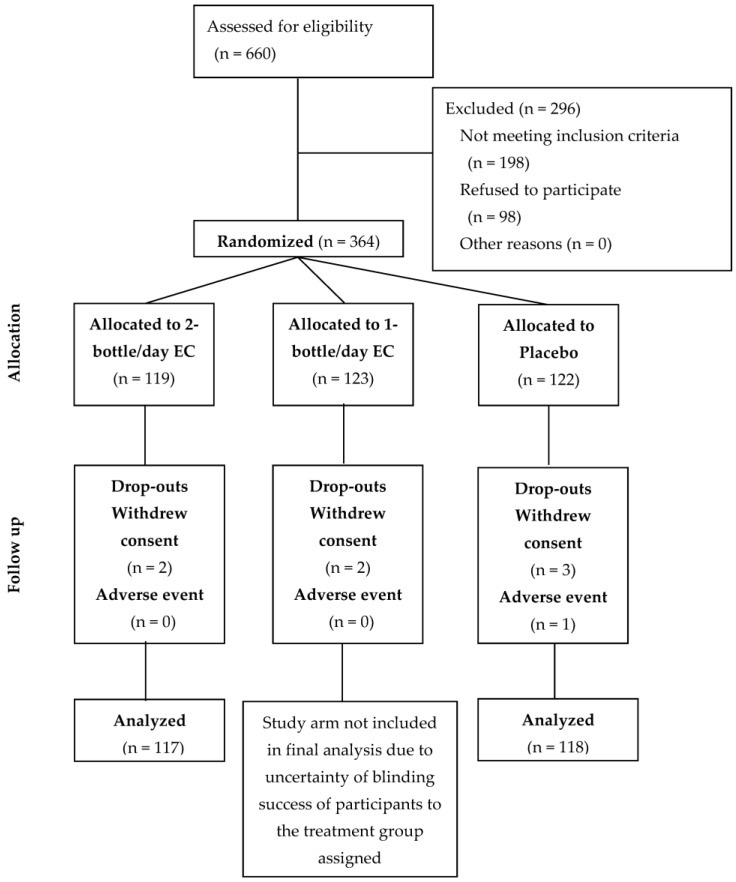
Participant flow diagram in the study based on the CONSORT flow diagram.

Eligible participants were recruited from March to October 2016 until the target sample size was achieved. In total, 119, 123 and 122 participants were allocated to the two bottles/day EC, one bottle/day EC and placebo groups, respectively. For reasons described in Section 2.6, only analyses of the two bottles/day EC (referred to as EC arm in the rest of the manuscript) and placebo arms are presented. Study dropout rate in both arms was low (*n* = 6, 2.5%), with two and four dropouts from the EC and placebo groups, respectively. Among the six dropouts, five withdrew consent and only one from the placebo group withdrew due to adverse events (diarrhea) (Figure 1). None of the participants was non-compliant to the treatment schedule, defined in the protocol as missed consumption of the investigational product for three or more consecutive days. Baseline characteristics including demographics, lifestyle and past medical conditions of participants in each group of the per-protocol set (*n* = 117, EC and *n* = 118, placebo) are presented in Table 2. 

EC and placebo groups were generally well-balanced on baseline characteristics shown in Table 2. The mean (SD) age of participants was 22.9 (±4.4) years and 22.7 (±4.8) years in the EC and placebo groups, respectively. More participants were female (69.2%, EC and 67.8%, placebo) and the highest education completed for the majority of participants was high school (68.4%, EC and 69.5%, placebo). There was no statistical significant difference in baseline demographic and lifestyle characteristics between the groups. The presence of past medical conditions was reported by a few participants, including type 2 diabetes mellitus (*n* = 1, EC and *n* = 2, placebo) and gastrointestinal disorder (*n* = 2, EC and *n* = 1, placebo). There was slight imbalance in baseline stress severity between the groups: more participants reporting “severe/extremely severe stress” in the EC group (12.7% vs. 7.69%) and more participants reporting “mild stress” in the placebo group (23.1% vs. 13.6%).

### 3.2. Cognitive Enhancing Effect of EC 

The primary analysis was conducted on 117 (98.3%) and 118 (96.7%) participants in the EC and placebo groups who completed the study, respectively. Baseline and change scores from baseline at 7 and 14 days of cognitive function tests are reported in Table 3. 

#### 3.2.1. Digit Span Forward

The three-way interaction effect (treatment × stress × visit) with *F* (3,227) = 3.34, *p* = 0.020, partial η2 = 0.042, was statistically significant, indicating that the two-way interaction effects between treatments and visits were different across stress subgroups. Baseline and change scores are reported by stress subgroups in Table 4. 

**Figure 2:**
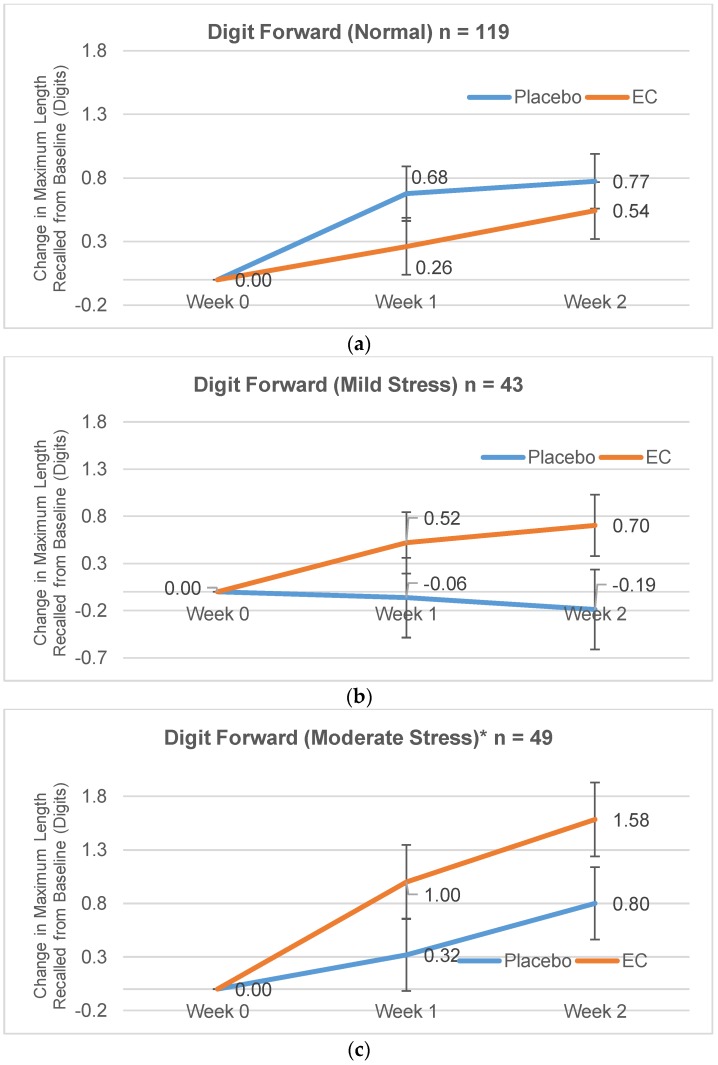

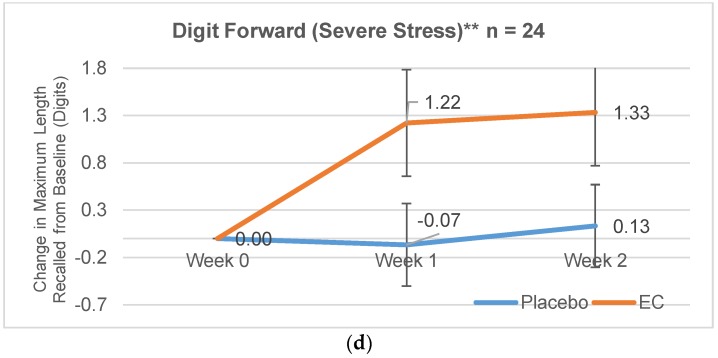
(**a**–**d**) Estimated Change in Maximum Length of Digits Recalled (±standard error) on the Digit Span Forward by Stress Subgroups at Week 1 and Week 2; * Statistically significant two-way (treatment × visit) interaction: *p* = 0.041; ** *p* = 0.065.

Two-way ANOVAs were conducted for each stress subgroup and the two-way interaction effect was significant only in the moderate stress subgroup, with *F* (1,47) = 4.42, *p* = 0.041, partial η2 = 0.086, and approaching statistical significance in the severe stress subgroup *F* (1,22) = 3.77, *p* = 0.065, partial η2 = 0.146. Graphically, Figure 2c shows that the EC group outperformed placebo group by around 0.70 digits (both at Day 7 and at Day 14) in the moderate stress subgroup. Figure 2a–d also shows that larger effect sizes (difference between EC and placebo) were observed in subgroups with higher baseline stress. Although the two-way interaction effect did not reach statistical significance in the severe stress subgroup, the small number of participants in this subgroup (*n* = 24) provided a low power to detect such a difference. In the normal and mild stress subgroups, two-way interaction effects were not significant with normal: *F* (1,117) = 1.44, *p* = 0.231, partial η2 = 0.012; and mild: *F* (1,41) = 2.36, *p* = 0.132, partial η2 = 0.055. 

There was some indication of baseline imbalance in Digit Span Forward scores in the stressed subgroups, with stressed placebo subgroups showing better baseline performance than stressed EC subgroups (Table 4). As such, we conducted a sensitivity analysis by excluding participants with baseline scores 9 or higher to explore if these differences could be due to greater ceiling effects in the placebo stressed subgroups which had higher mean baseline scores. However, sensitivity analysis showed that the two-way interaction (treatment × visit) term was statistically significant with *F* (2,146) = 5.44, *p* = 0.021, partial η2 = 0.036, indicating scores were different between the treatment groups across visits. Further analyses showed greater improvement in the EC group at Week 1 (mean difference: 0.71 digits; *p* = 0.047) and close to statistical significance at Week 2 (mean difference: 0.85 digits; *p* = 0.051). Thus, the benefit of EC detected in the primary analysis was found to be robust to the imbalance of baseline scores in the sensitivity analysis. 

#### 3.2.2. Digit Span Backward

**Figure 3:**
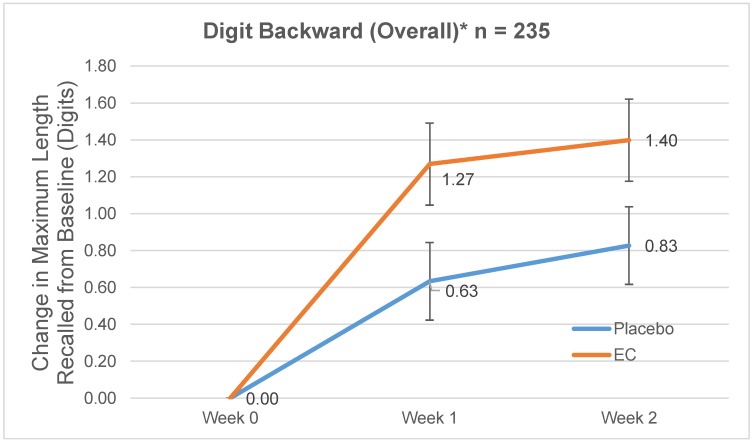
Estimated Change in Maximum Length of Digits Recalled (±standard error) on the Digit Span Backward at Weeks 1 and 2; * Statistically significant two-way (treatment × visit) interaction: *p* = 0.028.

The three-way interaction effect (treatment × stress × visit) in digit span backward was not statistically significant, with *F* (3,227) = 0.82, *p* = 0.479, partial η2 = 0.011. However, the two-way (treatment × visit) interaction effect was significant, with *F* (1,227) = 4.87, *p* = 0.028, partial η2 = 0.021. Figure 3 shows the estimated marginal means of the EC and placebo groups from the ANOVA model. EC group outperformed placebo by an estimated 0.60 digits at Day 7 and 0.50 digits at Day 14 (Figure 3).

#### 3.2.3. Stroop and SART Tests

**Figure 4:**
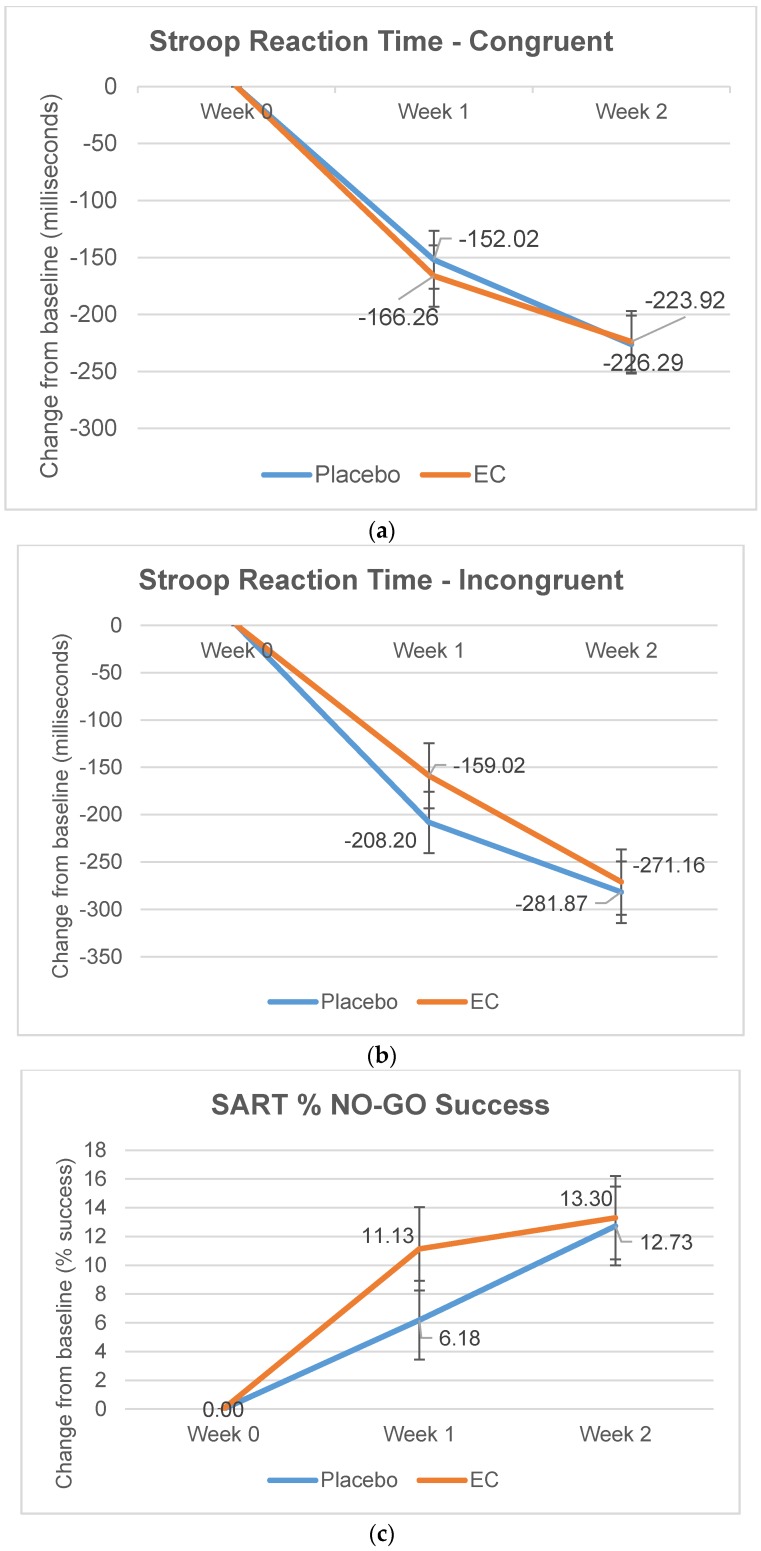

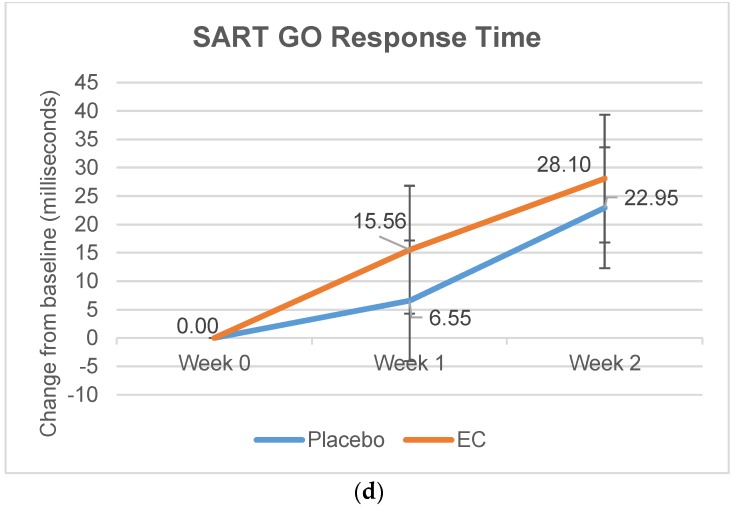
(**a**) Stroop reaction time—congruent; (**b**) Stroop reaction time—incongruent; (**c**) SART % NO-GO Success; and (**d**) SART GO Response Time. Estimated change in Stroop and SART test parameters (±standard error) with statistically significant visit effects at Weeks 1 and 2.

No statistically significant three-way (treatment × stress × visit) or two-way (treatment × visit) interaction effects were detected in any parameter of the Stroop and SART tasks. However, visit effects were significant in Stroop and SART tasks. For Stroop tasks, both EC and *p* groups improved reaction times in the congruent (*p* < 0.001) and incongruent (*p* < 0.001) conditions (Figure 4a,b). This may be due to practice effects previously shown in Stroop trials. [37] Both EC and *p* showed slower SART response times (*p* = 0.01; Figure 4c) but improved percentage no-go success in SART tasks (*p* = 0.01; Figure 4d). Practice effects on the SART have not been confirmed but a slight negative practice effect has been demonstrated, due to the tedious nature of the task, in a similar continuous performance test (CPT) for sustained attention [38].

### 3.4. Adverse Events

Similar proportions of subjects reported adverse events in the EC and placebo groups: *n* = 40 (33.6%) and *n* = 38 (31.1%), respectively. Adverse events were primarily mild in intensity and most events were assessed to be unrelated to the investigational products by study investigators. The proportions of adverse events that did not deny relationship with study product (i.e., unlikely, probable or certain relationship) were 10.6% and 11.3% in the EC and placebo groups, respectively. Among adverse events that were assessed to be of probable relation (i.e., probable and certain relationship) to investigational products, the most common events in participants were: diarrhea (*n* = 2, EC and *n* = 1, placebo), headache (*n* = 1, EC and *n* = 2, placebo) and nausea (*n* = 1, EC and *n* = 2, placebo). 

## 4. Discussion

This randomized, double-blind, placebo-controlled clinical trial demonstrated that EC consumption in healthy adult volunteers improved working memory, assessed by Digit Span Backward, during daily consumption for two weeks. EC also improved short-term memory, assessed by the Digit Span Forward, only among participants experiencing moderate or severe stress in daily life at baseline. No effect of EC consumption or subgroup effects from baseline stress levels was demonstrated in the domains of selective or sustained attention.

Our finding that EC improved working memory on the Digit Span test is largely consistent with earlier studies. In the meta-analysis of chicken extract on cognitive function, a pooled analysis of three studies using Digit Backward and overall Digit Span scores showed statistically significant beneficial effects of EC compared to placebo [16]. Previously, a brain function imaging study using near-infrared spectroscopy also showed statistically significant increases of oxy-hemoglobin concentrations in several prefrontal areas of the brain during a working memory task, indicating EC increased brain activity in these regions during mobilization of working memory [18].

In the domain of short-term memory, previous study findings are more varied. Our finding that EC improved short-term memory only in stressed participants, and to a larger extent with greater stress, supports the possibility that varied results could be due to heterogeneous populations studied earlier. Improvement with EC was shown in some studies [11,13,14] but not in others [15,17]. Interestingly, two of these studies showing improvement with EC consumption involved fourth-year medical students just before their examinations, who are likely to be highly stressed [10,13]. In another study [16] involving adults with reported work stress, only the subgroups with high anxiety or high depression scores consuming EC showed greater improvement in short-term memory than placebo but not in the overall study sample. Again, this supports the heterogeneity of EC’s effect on short-term memory. Since moderate to strong correlations have been demonstrated between stress and anxiety (*r* = 0.74) and stress and depression (*r* = 0.77) [39], it is likely that adults experiencing the related symptoms of stress, anxiety and depression show greater improvement in short-term memory with EC consumption.

Unlike short-term and working memory, EC showed no effect on selective or sustained attention in this study. This is interesting because the Digit Span Test itself is a measure of attention, in addition to encoding and auditory processing [40]. One possible explanation is that attention comprises of several semi-independent sub-systems, including selective attention, sustained attention, attentional switching, auditory-verbal working memory or divided attention [41,42]. Different sub-systems of attention are also known to respond differently to intervention in treatment efficacy studies [43]. Our findings showing the lack of effect of EC in selective or sustained sub-systems is generally consistent with earlier studies of EC on selective attention [8,15] and sustained attention [15,17]. Thus far, only a quicker reaction time on the Arrow–Flankers test for selective attention among participants consuming EC compared to placebo has been demonstrated [15]. No subgroup effect of baseline stress levels was found in either domain in our study. Unlike earlier RCTs of EC’s effect on cognitive function, [8,11,14,15,17], the current study reports results of a two bottles/day dose compared to a one bottle/day dose used in previous RCTs. Nonetheless, as summarized, results of the overall population in this study are generally consistent in all four domains when compared to earlier studies. The two bottles/day dose has also been studied previously but these studies have focused on recovery from mental fatigue [12,13] and functional brain blood blow imaging [18] of EC’s cognitive effects. 

During the two-week study period, visit effects were detected for Stroop and SART tasks (Figure 4a–d) with improvement observed in both EC and placebo groups over visits. Digit span performance also improved in both groups (Figure 2a,d and Figure 3) though visit effects were not statistically significant. The improvement seen in both groups could be due to practice effects [32,37] or overall nutrient intake with EC or placebo (marine collagen) consumption. Marine collagen peptides have previously shown neuroprotective effects in mice [44] and rats [45]. Nevertheless, the larger memory improvement seen with EC compared to placebo, despite slightly lower protein content, suggests the unique composition of bioactive peptides and amino acid complex in EC is associated with its cognitive benefits.

EC is rich in amino acids and the dipeptides, carnosine and anserine [1,4,15]. Carnosine and anserine have antioxidant and neuroprotective activity [46,47] and supplementation improved episodic memory [21,22,23] and mental status [24] among healthy middle-aged and elderly adults. EC also contains several amino acids [4] that have shown cognitive bioactivity when supplemented individually to the normal diet, such as tyrosine, histidine, glutamic acid, arginine, and the branched chain amino acids (BCAA). In healthy adults, tyrosine improved cognitive performance, especially in stressful conditions [48,49] and histidine shortened reaction time on a working memory task [50]. Glutamic acid improved learning and memory in rats [51] and arginine improved cognitive function in humans [52], showed antioxidant neuroprotective effects [53,54] and improved working memory [55] and spatial memory [53,55] in rats. BCAA supplementation has been studied in adult athletes and shown to improve cognitive function [56,57,58,59] due to modulation of neurotransmitter (serotonin and the catecholamines) synthesis and release [60,61]. As EC contains a complex of bioactive peptides and amino acids, with several components showing cognitive bioactivity, the memory enhancing effect seen in EC could be an overall effect of the complex rather than attributed to a single component. 

Improvements in short-term memory and working memory with EC consumption are important findings as both domains are highly regarded constructs in cognitive science and intellectual assessment [62]. Serial recall used in digit span tasks, is recognized as one of the fundamental human memory systems. It relies on an individual’s attention and capacity to maintain and recall objects in short-term memory. As the WAIS Digit Span is an auditory mode test, the Forward task measures attention, encoding and also auditory processing [63]. Although the Backward task appears to be very similar to the Forward task, different psychological processes are used [62]. The Backward task requires mental processing of information while simultaneously maintaining the digits in memory, thus is a measure of working memory capacity [40,63]. Digit Span Backward performance is also more strongly correlated with fluid intelligence (*r* = 0.55–0.58) than the Forward task [64,65]. Regardless, both the Forward and Backward tasks are correlated with general intelligence and are included as a test component of the Wechsler Adult Intelligence Scale (WAIS), which are the most widely used measures of intelligence in the world [62,63].

Our study is limited in the following ways: first, the relatively short two-week study duration does not elucidate longer-term effects of EC consumption. Secondly, physiological measurements such as serum cortisol or other biochemistry markers were not measured. Thus, though an effect was demonstrated, changes in physiological parameters and their correlation with observed effects that may explain mechanisms-of-action were not investigated in this study. However, previous clinical studies focused on elucidating specific mechanisms, as summarized in earlier paragraphs, support the clinical findings in this study [12,14,62].

As our study has shown heterogeneity of EC’s effect on short-term memory by baseline stress subgroups, future research could clarify if these are chronic effects mediated by stress reduction, using physiological stress markers such as serum cortisol. Other physiological and biochemical measurements associated with potential bioactives, such as carnosine and anserine, should also be taken to verify their efficacies or elucidate mechanisms of action. While earlier studies have shown improvement in acute recovery of cortisol levels with EC consumption [13,15], future research could particularly focus on chronic stress levels and its modulation with EC consumption, especially since chronic stress is known to adversely affect cognitive function [19].

## 5. Conclusions

EC improves the mental processes of working memory in healthy adults during daily consumption of EC for two weeks. EC also improves short-term memory among participants experiencing stress in daily life. Thus, healthy adults can experience memory benefits with short term consumption of EC, particularly when experiencing stress in daily life. Further research can investigate benefits on longer-term consumption of EC and clarify mechanisms and relationships between EC, stress and cognition. No effect in the attentional sub-systems of selective and sustained attention was found with EC consumption.

## Figures and Tables

**Table 1:** Nutritional content of study investigational products.

Content	Essence of Chicken (EC)	Placebo
Solid (g/100 g)	8.02	9.32
Protein (g/100 g)	7.51	8.10
Carbohydrate (g/100 g)	0.00	0.76
Fat (g/100 g) *	0.06	0.06
Ash (g/100 g)	0.58	0.40
Energy (Kcal/100 g)	30.58	35.98

* by acid hydrolysis.

**Table 2:** Participant demographic, lifestyle and past medical conditions recorded at baseline visit.

	Number (%) Unless Otherwise Stated	
Demographic and Baseline Characteristics	EC *n* = 117	Placebo *n* = 118
Age (years), mean (SD)	22.9 (4.4)	22.7(4.8)
Male	36 (30.8)	38 (31.2)
Female	81 (69.2)	80 (67.8)
Height (m), mean (SD)	1.63 (0.09)	1.65 (0.07)
Weight (kg), mean (SD)	54.8 (7.5)	55.9 (6.4)
BMI (kg/m^2^), Mean (SD)	20.3 (1.5)	20.5 (1.3)
Highest Education Completed		
Less than high school	0 (0.00)	1 (0.85)
High school	80 (68.4)	82 (69.5)
Diploma	4 (3.42)	0 (0.00)
University Degree	29 (24.8)	31 (26.3)
Masters’ Degree	4 (3.42)	4 (3.39)
Alcohol consumption		
None	94 (80.3)	91 (77.1)
1 drink per week	15 (12.8)	18 (15.3)
>1 drink per week	8 (6.8)	9 (7.63)
Currently smoking		
Yes	4 (3.42)	8 (6.78)
No	107 (96.6)	110 (93.2)
Past Medical Conditions *		
Diabetes Mellitus Type II	1 (0.85)	2 (1.69)
Elevated markers of renal insufficiency	1 (0.85)	0 (0.0)
Hypertension	0 (0.0)	1 (0.85)
Pelvic floor dysfunction	1 (0.85)	1 (0.85)
Pelvic or Abdominal surgery	2 (1.71)	1 (0.85)
Gastrointestinal disorder	2 (1.71)	1 (0.85)
Stress Category ^#^		
Normal	57 (48.7)	62 (52.5)
Mild	27 (23.1)	16 (13.6)
Moderate	24 (20.5)	25 (21.2)
Severe/Extremely Severe	9 (7.69)	15 (12.7)

^#^ According to the Depression Anxiety Stress Scale (DASS) Stress Scale Score definitions; * Self-reported past medical conditions: diabetes and hypertension reported as currently controlled without medication; Pelvic floor dysfunction, pelvic or abdominal surgery and gastrointestinal disorder reported as past conditions; and renal insufficiency reported as elevated blood urea nitrogen and creatinine in the past without diagnosis of renal insufficiency and currently normal values.

**Table 3:** Baseline and change scores of cognitive function test parameters at Days 7 and 14 in the Essence of Chicken (EC) and Placebo (P) groups.

Cognitive Function Test ^α^	Mean (SD) Score at Baseline		Mean (SD) Score Change from Baseline			
	EC *n* = 117	P *n* = 118	Day 7		Day 14	
			EC	P	EC	P
**WAIS Digit Span**						
Forward span	7.88 (1.66)	7.98 (1.51)	0.547 (1.53)	0.407 (1.58)	0.855 (1.84)	0.568 (1.86)
Backward span	7.06 (2.00)	7.16 (2.06)	1.12 (1.95)	0.78 (1.81)	1.34 (2.13)	1.08 (2.01)
**Stroop**						
Proportion Correct, Congruent	0.98 (0.03)	0.99 (0.03)	−0.005 (0.033)	−0.005 (0.039)	−0.007 (0.035)	−0.011 (0.041)
Proportion Correct, Incongruent	0.94 (0.08)	0.94 (0.07)	0.024 (0.077)	0.020 (0.072)	0.028 (0.079)	0.012 (0.081)
Reaction Time, Congruent	972.15 (278.02)	996.04 (350.55)	−134.85 (235.57)	−143.82 (237.90)	−204.96 (209.18)	−225.14 (265.94)
Reaction Time, Incongruent	1116.45 (326.09)	1173.97 (412.54)	−128.83 (316.55)	−202.99 (304.21)	−248.68 (262.49)	−272.52 (330.90)
Stoop Interference Index (ms)	144.30 (192.97)	177.93 (214.92)	6.02 (269.59)	−59.174 (257.97)	−43.71 (227.18)	−47.37 (218.06)
**SART**						
% No-Go Success	36.75 (26.92)	38.88 (27.22)	11.27 (21.28)	7.50 (26.48)	15.56 (24.84)	13.15 (28.36)
% No-Go Omissions	2.11 (2.94)	2.08 (4.38)	0.24 (3.83)	0.10 (4.43)	0.03 (4.64)	−0.13 (5.06)
Reaction Time, Mean (ms)	341.02 (92.62)	348.04 (92.44)	21.79 (94.10)	10.60 (98.88)	38.78 (108.43)	20.28 (96.11)

EC: Essence of Chicken; P: placebo; MD: Mean difference; ^α^ Test parameters: WAIS Digit Span Two-error Maximum Length of Digits; Stroop Reaction Time in milliseconds (ms); Stroop Interference Index calculated as Reaction Time of Incongruent−Congruent conditions; SART Reaction Time: Sustained Attention Response Task Mean Reaction Time in milliseconds (ms) of all tasks.

**Table 4:** Baseline and change scores of WAIS Digit Span Forward and Backward by Participant Stress Levels ^^^ in Essence of Chicken (EC) and Placebo (P) groups.

Cognitive Function Test ^α^	Mean (SD) Score at Baseline		Mean (SD) Score Change from Baseline			
	EC *n* = 117 ^#^	P *n* = 118 *	Day 7		Day 14	
			EC	P	EC	P
**Digit Span Forward**						
Normal	8.26 (1.75)	7.81 (1.56)	0.26 (1.62)	0.68 (1.50)	0.54 (1.65)	0.77 (1.93)
Mild Stress	7.89 (1.40)	8.19 (1.64)	0.52 (1.40)	−0.06 (1.77)	0.70 (2.03)	−0.19 (2.37)
Moderate Stress	7.33 (1.49)	8.04 (1.51)	1.00 (1.25)	0.32 (1.46)	1.58 (1.59)	0.80 (1.35)
Severe Stress	6.89 (1.62)	8.4 (1.18)	1.22 (1.79)	−0.07 (1.79)	1.33 (2.60)	0.13 (1.51)
**Digit Span Backward**						
Normal	7.05 (2.16)	6.94 (2.02)	1.16 (2.08)	1.02 (1.86)	1.49 (2.19)	1.39 (2.03)
Mild Stress	7.26 (2.11)	7.56 (2.22)	0.33 (1.90)	0.25 (1.57)	0.63 (1.80)	0.00 (2.10)
Moderate Stress	7.08 (1.64)	7.28 (2.20)	1.58 (1.41)	0.60 (1.91)	1.58 (2.30)	1.32 (2.04)
Severe Stress	6.44 (1.67)	7.47 (1.85)	2.0 (1.94)	0.67 (1.68)	1.89 (1.96)	0.60 (1.35)

EC: Essence of Chicken; P: placebo; MD: Mean difference; ^^^ According to the Depression Anxiety Stress Scale (DASS) Stress Scale Score definitions; ^α^ Test parameters: WAIS Digit Span Two-error Maximum Length of Digits; ^#^ EC (*n*, by DASS Stress Category): Normal (*n* = 57); Mild Stress (*n* = 27); Moderate Stress (*n* = 24); Severe Stress (*n* = 9); * *p* (*n*, by DASS Stress Category): Normal (*n* = 62); Mild Stress (*n* = 16); Moderate Stress (*n* = 25); Severe Stress (*n* = 15).
